# The Senior Companion Program Plus for African American Caregivers of Persons With Alzheimer Disease and Related Dementias: Protocol for a Randomized Controlled Trial

**DOI:** 10.2196/49679

**Published:** 2023-07-24

**Authors:** Noelle L Fields, Ling Xu, Ishan C Williams, Joseph E Gaugler, Daisha J Cipher

**Affiliations:** 1 School of Social Work University of Texas at Arlington Arlington, TX United States; 2 School of Nursing University of Virginia Charlottesville, VA United States; 3 School of Public Health University of Minnesota Minneapolis, MN United States; 4 College of Nursing and Health Innovation University of Texas at Arlington Arlington, TX United States

**Keywords:** Alzheimer disease, dementia, lay provider, senior companion, volunteer, intervention, culturally informed, African American, family caregivers

## Abstract

**Background:**

Alzheimer disease and related dementias (ADRD) pose significant challenges as chronic health conditions in the United States. Additionally, there are notable disparities in the diagnosis and prevalence of ADRD among diverse populations. Specifically, African American populations have a higher risk of developing late-onset ADRD than White people, and missed diagnoses of ADRD are more common among older African American populations than older White populations. These disparities also impact African American ADRD family caregivers.

**Objective:**

The overall goal of this project is to develop a culturally informed, lay provider psychoeducational intervention named Senior Companion Program Plus (SCP Plus), which is specifically designed for African American ADRD caregivers and is potentially accessible, affordable, and sustainable.

**Methods:**

In the proposed explanatory sequential mixed methods study, a randomized controlled trial will be used that includes 114 African American family caregivers of a relative with ADRD who will participate in the 3-month SCP Plus program.

**Results:**

The study was funded on September 15, 2018, by the National Institutes of Health (1R15AG058182-01A1). Data collection began on May 16, 2019, but due to COVID-19 restrictions, ended 12 months into the planned 27-month recruitment period on March 31, 2023. The study was completed in June 30, 2023, and currently the results are being analyzed.

**Conclusions:**

The SCP Plus offers promise as an intervention that utilizes an existing platform for the delivery of a lay provider intervention and offers a novel approach for addressing gaps in accessible, community-based support for caregivers of people with ADRD.

**Trial Registration:**

ClinicalTrials.gov NCT03602391; https://classic.clinicaltrials.gov/ct2/show/NCT03602391

**International Registered Report Identifier (IRRID):**

RR1-10.2196/49679

## Introduction

### Background

Approximately 6.7 million Americans aged 65 years and older are currently impacted by Alzheimer disease and related dementias (ADRD) [1]. Given that age is a key risk factor for ADRD and the number of older Americans continues to grow, it is expected that cases of ADRD will also increase [1]. ADRD is a disease marked by profound disability and dependence that worsen over time. Informal family caregivers provide most of the care to persons with ADRD, and they are at greater risk for negative physical and psychosocial outcomes when compared to noncaregivers or other types of caregivers [2].

Several intervention strategies have been implemented and evaluated to alleviate stressors and other negative outcomes among dementia family caregivers. In particular, research suggests that psychoeducational interventions may positively impact caregiver well-being, including burden [3], perceived health [4], and mental health [5,6]. While most psychoeducational interventions use trained interventionists, [7,8] peer-led interventions have demonstrated considerable promise. For example, research suggests that peer-led interventions may enhance family caregivers’ social support and help with the management of chronic conditions [9-11]. Additionally, the translation potential of peer-led programs is further enhanced because of the use of lay providers rather than trained interventionists.

### African American ADRD Caregivers

Based on demographic projections, the African American older population will grow to nearly 11% of the older adult population in the United States by 2050 [12]. Compared to White people, African Americans are about 2 times more likely to develop ADRD [1] and may live longer with ADRD than other races [13]. However, there are disparities in how ADRD is diagnosed and treated among African American older adults. For example, research suggests that missed diagnoses of ADRD occur more often among older African American adults than among Whites [14].

These disparities also affect African American ADRD family caregivers. Among this group, outcomes related to caregiver burden are mixed. For example, as reported by Brewster et al [15], a study presented by McLennon [16] indicated low scores on the Zarit Burden Scale [17] in African American ADRD family caregivers of persons with moderate-to-severe ADRD. However, the qualitative findings suggested that ADRD family caregivers experienced several challenges related to finances, safety concerns such as wandering, and providing physical care. Similarly, Brewster and colleagues [15] reported that in a mixed methods study of African American ADRD family caregivers conducted by Moss et al, participants scored relatively high on measures of quality of life but qualitatively reported stress and negative aspects related to caregiving [18]. Although psychosocial interventions for caregivers of persons with ADRD have shown promise, more research on culturally relevant interventions for African American ADRD caregivers is needed.

### The Senior Companion Program

The Senior Companion Program (SCP) is one of 3 core programs funded by AmeriCorps.The SCP focuses on lower-income older adult volunteers providing assistance and friendship to other older adults who need support with daily living tasks [19]. SCP volunteers must be “at least 55 years of age or older and be capable of serving the frail elderly or adults who have one or more physical, emotional, or mental health limitations and are in need of assistance to achieve and maintain their highest level of independent living” [20]. Senior companions (SCs) serve as volunteers for 5 to 40 hours per week and receive modest hourly stipends [20]. Their typical tasks include providing assistance with meal preparation, offering medication reminders, accompanying their care recipient (CR) to social activities and medical appointments, and providing respite to family caregivers [20].

### The Senior Companion Program Plus

Despite the benefits of the services provided by the SCP, many ADRD caregivers still report significant stress, as the SCP does not currently include a unique service to address caregiver stress/burden for ADRD caregivers [21]. Augmenting the SCP with ADRD-specific training is ideal as it extends the typical services of the SCP by providing psychoeducation to address the stress and burden that ADRD family caregivers continue to experience.

The Senior Companion Program Plus (SCP Plus) is the proposed intervention adapted from the work of Morano and King [22]. However, the SCP Plus differs from that work by training lay providers (ie, SCs) rather than clinical experts to deliver the intervention to increase its accessibility and translational potential. In the pilot study of the SCP Plus [21,23], the research team held focus groups with African American SCs to refine the work of Morano and King [22] and to adapt their intervention for use with the SCP. The SCP Plus trains SCs as lay provider “interventionists” to deliver a 9-week in-home psychoeducational intervention with African American ADRD caregivers. The SCP Plus includes 9 educational modules that cover topics on education, safety, strategies for challenging behaviors, communication, coping, finding purpose/meaning in caregiving, and community-based services (for an overview of each module, see Fields et al [23]).

The SCP Plus includes 12 hours of training for the SCs over 2 days and reviews the essential components of the intervention. Following this training, SCs deliver 1 module of the SCP Plus to the African American dementia caregivers during a weekly 60-minute meeting in the caregivers’ or CRs’ homes over a 3-month period (for a total of 9 hours). Based on the success of a pilot study of the SCP Plus [21,23], the proposed project will expand the program to 3 US states to conduct a randomized evaluation of efficacy.

For this proposed study, the revised sociocultural stress and coping model [24,25] provides the conceptual framework to examine caregiver burden, along with how cultural values affect the coping style, social support, and well-being of ADRD caregivers. This study’s aims and hypotheses are listed in Textbox 1.

**Study aims and hypotheses.**

- Aim 1: We will examine the specific effects of the Senior Companion Program Plus (SCP Plus) to increase knowledge about Alzheimer disease, reduce caregiver burden and stress, and increase caregiver well-being after the SCP Plus intervention.
- Hypothesis 1: Participants will indicate clinically significant and statistically significant (*P*<.05) reductions in caregiver burden/stress and increased caregiver well-being at 3 months and a 6-month postintervention interval.
- Aim 2: We will examine the specific effects of the SCP Plus to improve caregiver coping skills.
- Hypothesis 2: Participants will indicate statistically significant (*P*<.05) improvements in coping skills associated with dementia caregiving.
- Aim 3: We will analyze the specific effects of the SCP Plus to increase caregiver satisfaction with social support (eg, received support, satisfaction with support, social support network, and negative interactions).
- Hypothesis 3: Participants will indicate statistically significant (*P*<.05) increases in social support.
- Aim 4: We will explore and interpret the statistical results obtained in the first quantitative phase to help explain why participants who scored in the lower and upper quartiles on caregiver burden/stress were or were not impacted by the usefulness of the intervention.

## Methods

### Study Design

The proposed study is a randomized control trial that will assess the effects of SCP Plus on African American dementia caregiver stress and burden, coping skills, and social support. An explanatory sequential mixed methods design [26] will be utilized, involving collecting quantitative data first and then analyzing the data to identify subsamples of participants who reported the greatest and least change in caregiver stress/burden. Among individuals in the SCP group with the highest quartile (n=14, 25%) and lowest quartile (n=14, 25%) of change, we will conduct follow-up qualitative interviews to capture the details of their situation and present a more comprehensive analysis that leverages the strengths of both methods. We propose a 3-year study. The study will take place in collaboration with SCP at sites in Texas, Louisiana, and Arkansas with approximately 114 SC-caregiver dyads. Half of the sample will receive services as usual (SAU) without the SCP Plus intervention. The control group will be randomly selected after baseline screening of all family caregivers, and all measures will be collected blind to the group condition. To further prevent selection bias, statistical techniques such as mixed factorial analysis of covariance (ANCOVA) will be conducted to adjust for covariate imbalance during data analysis.

### Participants and Setting

Power analyses performed with G*Power software (version 3.1.9) indicated that a total of 114 participants are required to address our primary research objective using a mixed factorial ANOVA of our primary study outcome—caregiver burden. This sample size estimate was based on a small effect size (*f*=.12) and a 2-tailed alpha of .05 and a beta of .20. This effect size estimate was generated from our pilot data, which indicated a mean change in caregiver burden of 2.82.

Study participants will be the SC-caregiver dyads that include the SCs (n=114) recruited from the state-wide agencies with SCP as well as the African American caregivers (n=114) of older adults with ADRD who are currently enrolled in a Senior Companion Program in Texas, Arkansas, or Louisiana. Each dyad consists of 1 SC matched with 1 caregiver. To be eligible for this study, SCs must be (1) currently participating in the SCP and (2) currently providing respite services to the caregivers. As per the requirements of the SCP, SCs must be “55 years of age or older, be determined by a physical examination to be capable of serving the frail elderly, and be able to provide such service without detriment to either themselves or the clients served” [20]. For caregivers that receive services from the SCs, they must be (1) self-identified as African American, (2) 21 years of age and older, (3) providing unpaid care for an older adult with ADRD, and (4) cognitively intact as measured by a 6-item screener [27]. CRs with ADRD must have a physician diagnosis of ADRD and live at home in the community. Caregivers who are involved in another caregiver psychosocial intervention study or have an acute illness that would prevent them from participating for at least 6 months will not be eligible.

### Study Procedures

#### Randomization

The SCP directors at each site will first help identify the potential SC-caregiver dyads who meet the inclusion criteria. The identified potential participants will then be randomly selected, and the research team will contact them afterward. Participant dyads who are interested in participating in the study will be guided through the informed consent process, administered a baseline survey via phone, and randomized to one of two groups: (1) the SCP Plus group that will receive the intervention plus SAU and (2) the control group that only receives SAU as part of the SC Program. SAU includes providing respite care for the family caregiver and personal care and light housekeeping/meal preparation for the person with dementia.

#### Data Collection

Quantitative data will be collected at preintervention, postintervention, and at the 6-month postintervention follow-up. The research team will then analyze the quantitative data at 3 time points to assess the effectiveness of the intervention and identify caregivers who reported the greatest and least change in caregiver stress/burden. Individuals in the highest and lowest quartiles of change in stress/burden will be recruited for follow-up interviews to understand their results in more depth through a qualitative study analysis.

#### SAU Program

For SAU, SCs volunteer with frail, low-income older adults approximately 20 hours a week. SCs’ tasks include helping older adults with ADRD by accompanying them to health care professional appointments, accompanying them to recreation/social activity/senior centers; providing assistance with ambulation and mobility; dressing, grooming, and feeding; reading, writing letters/forms; light cleaning; light meal preparation; monitoring for safety; peer support/companionship; and general caregiver respite. As part of the SCP, all SCs receive 40 hours of training from their agency, which includes topics such as program and agency policies, code of conduct, and a criminal background check.

#### SCP Plus

SCs randomly assigned to the SCP Plus will receive the 12-hour training. Two members of the research team will conduct all the SCP Plus training in person at each site. The training protocol was developed to affirm and build on the SCs’ existing knowledge and skills related to ADRD caregiving [23]. The SCP Plus training will be delivered in person in a group format that includes both didactic approaches and role-play experiences. The SCP Plus training will last 2 days (6 hours per day). To ensure treatment fidelity related to the training, manuals and protocols for the training were created before the onset of this study. The training material will be audio recorded and reviewed by the research team using a checklist to quantify adherence to the training program.

SCP Plus is based on a dementia caregiver program [22] and adapted by Fields and colleagues [21,23]. ADRD caregivers whose SCs are randomly assigned to the intervention group will receive the SCP Plus intervention. The SCP Plus intervention will be delivered face-to-face by the SC who is a part of the SC-caregiver dyad. The intervention involves 9 in-home psychoeducational sessions over a 3-month period with 1 session per week.

Every week for 9 weeks, the SC-caregiver dyad will meet for 1 hour in the home of the CR (ie, the person with dementia). This allows the SC to maintain their duties while still participating in the SCP Plus program. It also allows the ADRD caregiver to receive intervention in the home setting of the person with dementia. Each week, the SC will deliver 1 module from the SCP Plus. “Icebreaker” questions will be included at the beginning of each module to answer any questions that the ADRD may have from the previous module and to facilitate a time for informal, supportive communication between the SC and the ADRD caregiver. At the end of each module, the SC will ask the ADRD caregiver to reflect on the content of the module, which will be written down in the form of brief notes by the SC. The inclusion of incentives (ie, retail gift cards) will be used to reduce caregiver attrition.

### Measurements

The outcome evaluation will include measures of caregivers’ stress and burden, knowledge of ADRD, coping skills, social support and activity, caregiver appraisal, caregiver physical health, and cultural justifications for caregiving. Standardized measures will be used to assess these outcome variables. The study by Xu and colleagues [28] contains an illustration of how these measures are mapped onto the conceptual model for this study.

The sociodemographic characteristics of the caregivers and their CRs will be collected at baseline, including age, gender, education, length of caregiving relationship, self-rated health [29], and number of hours spent in caregiving. Information about CRs’ activities of daily living (ADL) and instrumental activities of daily living (IADL) that require help from caregivers will also be collected [30]. The Global Deterioration Scale [31] will be used to measure the severity of dementia of the person with ADRD. The frequency and duration of weekly contact with the SC will also be collected from the agency timesheets.

Dementia Caregiver burden and stress will be measured by the Zarit Burden Interview (ZBI) [17]. The ZBI was developed to measure the burden experienced by caregivers of persons with dementia living in the community [32]. The ZBI has 22 items measured with a 5-point Likert scale ranging from (never) to 4 (nearly always). Total sum scores range from 0 to 88, with higher scores indicating higher levels of distress. Studies have shown that ZBI has good validity and reliability among various minority dementia caregivers [33], particularly African American caregivers [34].

Knowledge of ADRD will be measured by the Knowledge of Dementia (KAD) scale [35]. KAD consists of 4 subscales concerning the (1) epidemiology and etiology of ADRD, (2) perceived effectiveness of different and currently available treatments, (3) beliefs regarding the perceived threat of ADRD for oneself, and (4) how respondents learned about the disease. KAD has shown good reliability and validity in assessing attitudes and beliefs regarding ADRD among dementia caregivers of different ethnic minorities [36].

Coping skills will be measured by the Brief Coping Orientation to Problems Experienced (COPE) subscales and religiosity coping. The Brief COPE questionnaire consists of 28 items measuring the ways/strategies caregivers have been coping with the stress in their life with a 4-point scale ranging from 1 (“I have not been doing this at all”) to 4 (“I have been doing this a lot”). This scale has shown good validity and reliability in caregivers of persons with dementia [37]. Religiosity coping will be examined with the short form of the Brief Religious Coping (RCOPE) scale to measure the positive and negative aspects of religious coping [38]. All items are measured on a 4-point scale ranging from 0 (“a great deal”) to 3 (“not at all”). This measure has shown good validity and internal consistency among racially and ethnically diverse populations of ADRD caregivers [33], including African American participants [39].

Social support will be measured with 13 items from four domains: (1) received support [40,41], (2) satisfaction with support [41,42], (3) social support network [43], and (5) negative interactions [41]. Social support networks will be measured by a modified Lubben Social Network Scale [43] that uses 3 items on a 6-point scale (none, 1, 2, 3 or 4, 5 to 8, and 9 or more). All other items have a 4-point scale that ranges from 0 (never) to 3 (very often) [44]. Social support measures have been proven to be valid and reliable with ethnic minority dementia caregivers [39], including African American dementia caregivers [39].

Caregiver appraisal will be measured with two components: (1) appraisal of problem behaviors and (2) appraisal of benefits. For an appraisal of problem behaviors, a modified version of the Revised Memory and Behavior Problems Checklist (RMBPC) [45] will be used to ask caregivers whether CRs’ problems occurred during the past week (yes or no). If the answer is yes, caregivers will then be asked to rate how much the problem “bothered or upset” them on a 5-point Likert scale ranging from 0 (not at all) to 4 (extremely). RMBPC has been shown in the literature to be a valid and reliable measure for different ethnic minority caregivers [46], especially African American caregivers [47]. For appraisals of benefits from caregiving, the Positive Aspects of Caregiving (PAC) scale will be assessed [48]. The PAC has 9 statements about the caregiver’s mental affective state regarding their caregiving experience. Each statement begins with “Providing help to the care recipient has…” followed by specific items (eg, made me feel useful, enabled me to appreciate life more). Each statement is rated on a 5-point Likert scale ranging from 1 (disagree a lot) to 5 (agree a lot). Studies show that the PAC is a valid and reliable measure for different ethnic minority dementia caregivers [33] and especially useful for African American dementia caregivers [49].

Caregiver well-being will be measured by the 12-Item Short-Form Health Survey (SF-12) [50] and the 16-item short version of the Caregiver Well-Being Scale (CWBS-ShortVersion). The SF-12 is a frequently used screening scale used to measure physical and mental health outcomes. It asks participants about their health, how they feel, and how well they can complete their usual daily activities. It has been shown to be a valid and reliable measure with African American older adults [51]. The CWBS-ShortVersion was developed to help family caregivers, clinicians, and researchers to identify areas of caregiver strength and areas in which additional support is needed. All 16 items will be measured by a 5-point scale ranging from 1 (rarely) to 5 (usually). The CWBS-ShortVersion has good content and construct validity regardless of the ethnicity of the caregivers [52].

Cultural justifications for caregiving will be measured by the Cultural Justifications for Caregiving Scale (CJCS) [53]. The CJCS consists of 10 items designed to assess caregivers’ cultural reasons and expectations in providing care. Each item is measured using a 4-point Likert scale ranging from 1 (strongly disagree) to 4 (strongly agree). Summed scores range from 10 to 40, with higher scores indicating stronger cultural reasons for giving care. Study results have shown this scales’ good reliability and validity score with African American caregivers [54].

### Qualitative Questions

The second qualitative phase will explore and interpret the statistical results obtained in the first quantitative phase. The interview protocol will include a semistructured interview guide to explore domains of difference (eg, perceptions of treatment, level of engagement, and intervention processes).

### Data Analysis

#### Quantitative Data Analysis

After the data are collected and entered into statistical software, the data quality will be carefully reviewed and then checked both automatically and manually. These review processes include checking the coding of observations, responses, and out-of-range values in SPSS software (IBM Corp), adding variable and value labels where appropriate, checking completeness or any errors of data entry, double-checking any errors in statistical analyses, and correcting errors made during transcriptions. In addition, data will be reviewed for risk of disclosure of research participants’ identities, for sensitive data, and for private information to ensure confidentiality. Since all the key concepts in the proposed study are measured with standard scales, reliability tests for each scale and test-retest reliability across the 3 surveys will be conducted. Summed scores will be calculated for each of the standard scales.

Any random missing data, such as those due to random mistakes in data collection and entry, and are not related to research staff or patient noncompliance, will be submitted to maximum likelihood estimation procedures to estimate those missing values. Maximum likelihood estimation for random missing data is a common and accepted procedure for missing data estimation [55,56].

We will apply an intent-to-treat analytic approach to the study data. Fisher exact tests, Pearson chi-square tests, and independent sample *t* tests will be computed to test for group differences that may be attributable to caregiver variables such as gender, age, ethnicity, clinical site, length of caregiving relationship, and number of hours spent in caregiving. If the groups significantly differ on any of these variables, the variable will be incorporated into the following analyses as a covariate, thereby controlling for potential influences that the covariate may have on the dependent variables. As noted in the statistical literature, large power gains can be achieved by including correlated stationary covariates in ANCOVA, ordinary least squares regression, and Cox proportional hazards regression [57]. Moreover, if at the culmination of this study, our sample sizes are unequal in the treatment groups and our outcome data are nonnormal, we will follow through with appropriate statistical corrections where necessary.

Mixed factorial ANCOVAs will be computed to compare the SCP Plus and SAU over time on caregiver burden (ZBI), beliefs about dementia (KAD), coping (Brief COPE), social support, caregiver appraisal, well-being (CWBS-ShortVersion), and cultural expectations (CJCS). The covariates will be any significant variable yielded from the univariate analyses, and the dependent variable will be the scores at the pretest, posttest, and 6-month follow-up periods. Moreover, 95% CIs will be computed for each adjusted mean difference. The study alpha will be set at .05, with no adjustments for multiple comparisons due to the preliminary nature of this study and the increased risk of type 2 errors as a result of alpha adjustment [58]

#### Qualitative Data Analysis

Qualitative interviews will be recorded digitally, transcribed, and analyzed using conventional content analysis [59]. A constant comparative approach [60] will be used to explore similarities and differences in participants who did and did not benefit from the SCP Plus. Multiple strategies will be utilized to address rigor and credibility including peer debriefing and an audit trail.

### Ethics Approval

The study protocol was approved by the Institutional Review Board of the University of Texas at Arlington (2017-0431 on 9/30/2018). It will be conducted following the Declaration of Helsinki.

## Results

This study was funded on September 15, 2018, by the National Institutes of Health (1R15AG058182-01A1). Data collection began on May 16, 2019, and ended on March 31, 2023. The study was completed in June 30, 2023, and currently the results are being analyzed.

The study participants are SC-caregiver dyads. Due to COVID-19 social distancing requirements, data collections were halted in March 2020, 12 months into the planned 27-month recruitment period. The final sample size for the proposed study consisted of 20 dyads at baseline, and only 11 CGs completed the posttest and follow-up test. There were no missing data, so imputation was not necessary.

For the SCs in the 3 sites (N=20), all of them were female with a median age of 71 (range 58-80) years. Over one-third were either widowed or divorced (n=8, 40%). In terms of educational status, half (n=10, 50%) of the SCs were high school graduates or below. All SCs reported attending a religious service at least once per month. The participating SCs had taken care of their current CR with ADRD for a median of 4.5 (range 1-15) years and provided a median of 30 (range 6-40) hours weekly for their CRs. The majority (n=17, 85%) of the SCs felt very confident or confident in providing care to their clients with ADRD. Half (n=10, 50%) of them reported difficulty in paying for their basic needs (ie, financial strain). In general, the SCs had moderate levels of self-rated health (median 3). They helped their CRs with ADL (median 2.5, range 0-6) and IADL (median 7.5, range 4-8).

For the caregiver participants, the 20 CGs were a median age of 60.5 (41-80) years. The majority were female (n=17, 85%), married (n=11, 55%), and lived with their CR (n=16, 80%) for a median of 3 (range 0-48) years. The majority (n=15, 75%) of the CGs worked outside of the home, 45% (n=9) were employed full-time, and 35% (n=7) had to reduce their working hours to take care of the CRs. The CGs reported that spirituality was important (median 3.5, range 1-4). They also regularly attended religious services (median 3, range 1-5) and often prayed or mediated (median 5, range 1-5). The majority (n=13, 65%) of the CGs were children of the CRs. They had been taking care of their CRs for a median of 5 (range 1-26) years and provided a median of 12 (range 2-24) hours of care daily. The majority of CGs used formal services “several times a week” (n=16, 80%) and were “satisfied” or “strongly satisfied” (n=18, 90%) with the services received. The CGs had moderate self-rated health (median 3, range 1-5). They reported their CRs’ general health as low (median 1.5, range 1-4) and their memory issues as a median of 25 (range 10-49).

For other results, please see Xu and colleagues [28].

## Discussion

### Expected Findings

SCP Plus offers an innovative approach to using a peer-led psychoeducational intervention for caregivers of persons with ADRD. Given that the SCP is a national program, there is potential for sustainability and cost-effectiveness. SCP Plus will advance the scientific knowledge about how African American ADRD caregivers can build coping skills and protective factors through engagement with lay health care providers such as SCs. The implementation of the SCP Plus will increase insight into the sociocultural experiences of dementia caregivers and enhance the ability of community-based providers to meet the unique needs of minority caregiver populations.

### Limitations

Due to the risks and feasibility of conducting an in-person intervention with SCs and ADRD caregivers during COVID-19, data collection for the intervention was suspended in March 2020. For a full description of the study limitations, see Xu et al [28].

### Comparison With Prior Work

During the past 30 years, the development and evaluation of ADRD caregiver interventions have focused on providing information, skills building and psychoeducation, psychosocial support, and respite to families [61]. Although many ADRD caregiving interventions have demonstrated efficacy, these programs are inaccessible to the majority of family caregivers; for these reasons, current efforts have focused on translating interventions into clinical or community-based settings to evaluate the relevance of study outcomes for key stakeholders [62,63]. However, it remains unknown whether existing dementia caregiver interventions are culturally relevant to African American dementia caregivers [62].

### Conclusions

The proposed project is aligned with the National Alzheimer’s Project Act [64] to better address the needs of persons with ADRD and their family caregivers. In particular, SCP Plus is aligned with three NAPA recommendations: (1) ensuring that family and unpaid caregivers have the support that they need; (2) ensuring representation and diversity in ADRD research; and (3) researching evidence-based interventions for ADRD care support. Finally, the implementation of SCP Plus will increase knowledge about how ADRD caregivers from minority populations can build coping skills and protective factors through engagement with lay health care providers such as SCs.

## Appendix

Multimedia Appendix 1 Peer-review reports from the Center for Scientific Review Special Emphasis Panel, Nursing and Related Clinical Sciences, National Institutes of Health (USA).

## Abbreviations

ADL: activities of daily living
ADRD: Alzheimer disease and related dementia
ANCOVA: analysis of covariance
CJCS: Cultural Justifications for Caregiving Scale
COPE: Coping Orientation to Problems Experienced
CR: care recipient
CWBS-ShortVersion: Short Version of the Caregiver Well-Being Scale
IADL: instrumental activities of daily living
KAD: Knowledge of Dementia
PAC: Positive Aspects of Caregiving
RCOPE: Brief Religious Coping
RMBPC: Revised Memory and Behavior Problems Checklist
SAU: services as usual
SC: Senior companion
SCP Plus: Senior Companion Program Plus
SCP: Senior Companion Program
SF-12: 12-Item Short-Form Health Survey
ZBI: Zarit Burden Interview

## Data Availability

This research does not use a publicly available data set.
