# Supplementary material for: The Senior Companion Program Plus for African American Caregivers of Persons With Alzheimer Disease and Related Dementias: Protocol for a Randomized Controlled Trial
Source: JMIR Res Protoc. 2023 Jul 24;12:e49679. doi: 10.2196/49679 (PMC10407770; doi:10.2196/49679)
Supplement: Multimedia Appendix 1 [file resprot_v12i1e49679_app1.pdf]

SUMMARY STATEMENT
(Privileged Communication)

PROGRAM CONTACT:
LISA ONKEN
(30l) 496-3131
lonken@nia.nih.gov

Release Date: **03/02/2018**
Revised Date: **03/05/2018**

Application Number: **1 R15 AG058182-01A1**

Principal Investigators (Listed Alphabetically):

FIELDS, NOELLE (Contact)
XU, LING

Applicant Organization: UNIVERSITY OF TEXAS ARLINGTON

Review Group: **ZRG1 NRCS-V (08)**
Center for Scientific Review Special Emphasis Panel
Nursing and Related Clinical Sciences

Meeting Date: **02/08/2018**
Council: **MAY 2018**
Requested Start: **07/01/2018**

RFA/PA: **PA16-200**
PCC: **2BBCHLO**
Dual IC(s): **NR**

Project Title: **The Senior Companion Program Plus (SCP Plus): A Psychoeducational Intervention for African American Dementia Caregivers**
SRG Action: **Impact Score:38 Percentile:18 +**
Next Steps: **Visit https://grants.nih.gov/grants/next_steps.htm**
Human Subjects: 48-At time of award, restrictions will apply
Animal Subjects: 10-No live vertebrate animals involved for competing appl.
Gender: 1A-Both genders, scientifically acceptable
Minority: 2A-Only minorities, scientifically acceptable
Children: 3A-No children included, scientifically acceptable
Clinical Research - not NIH-defined Phase III Trial

Project Year	Direct Costs Requested	Estimated Total Cost
1	299,017	459,993
TOTAL	299,017	459,993

ADMINISTRATIVE BUDGET NOTE: The budget shown is the requested budget and has not been adjusted to reflect any recommendations made by reviewers. If an award is planned, the costs will be calculated by Institute grants management staff based on the recommendations outlined below in the COMMITTEE BUDGET RECOMMENDATIONS section.

1R15AG058182-01A1 Fields, Noelle

PROTECTION OF HUMAN SUBJECTS UNACCEPTABLE

RESUME AND SUMMARY OF DISCUSSION: The application proposes a project to develop an in-home psychoeducational intervention (The Senior Companion Program Plus, or SCP Plus) for African American Alzheimer's disease and related dementia (ADRD) caregivers who are particularly affected by poverty, discrimination, and barriers to health care services and supports. The investigators plan to train senior companions to provide the intervention in the homes of the African American ADRD caregivers, and will conduct a randomized trial of SCP Plus. The application addresses a significant need for an accessible and cost-effective intervention in an underserved population. It is based on a premise that recognizes the needs of the population and African American caregiver coping styles. The PI and team are outstanding; in response to the prior review a biostatistician was added. The research environment is also excellent. Most reviewers found the application to be novel in its approach, but not all agreed that another psychoeducational study is either innovative or significant. Among the design strengths are attention to sampling, fidelity, training procedures, and well-supported measures. However, there are also a number of threats to the rigor of the study including the comparison group selected, the power analysis, training of data collectors, and lack of a true mixed methods analysis plan. Concerns were raised regarding the small number of students included, and the distances across sites. While reviewers weighed strengths and weaknesses somewhat differently, upon discussion they concurred that the application is likely to be of moderate to high impact as an Academic Research Enhancement Award.

DESCRIPTION (provided by applicant): Alzheimer's disease and related dementia (ADRD) is one of the most challenging chronic health conditions in the United States and considerable disparities exist in the diagnosis and prevalence of ADRD among communities of color. Research suggests that ADRD caregiver interventions have demonstrated efficacy, however, it remains unknown whether existing ADRD caregiver interventions are useful or accessible to African American ADRD caregivers in community settings. The primary goal of the proposed project is to develop an in-home psychoeducational intervention (The Senior Companion Program Plus, or SCP Plus) that is accessible, sustainable, and affordable for African American ADRD caregivers. The SCP Plus will focus on African American ADRD caregivers who are particularly affected by poverty, discrimination, and barriers to health care services and supports. A recent pilot study has established the feasibility and utility of SCP Plus. With the assistance of 6 student research assistants over the course of the project, we will implement the SCP Plus at sites in Texas, Louisiana, and Arkansas, recruiting approximately 114 participants. The participants will be randomized with 57 caregivers receiving the SCP Plus and 57 caregivers receiving services as usual with the Senior Companion Program. A weekly, 60 minute psychoeducational module will be delivered face-to-face across 9 weeks and will focus on education about ADRD, behavior management, communication skills, and aspects of providing care that enhance meaning such as spirituality. In order to provide an accessible and cost effective intervention that is potentially sustainable, senior companions will be trained to provide the intervention in the homes of the African American ADRD caregivers. Specific Aim 1. Determine whether SCP Plus reduces level of burden and stress among African American caregivers over a 3- and 6-month period when compared to a usual care control group. Specific Aim 2. Ascertain if SCP Plus improves coping skills among African American caregivers over a 3- and 6-month period when compared to a usual care control group. Specific Aim 3. Examine whether SCP Plus improves the level of satisfaction with support social among African American caregivers over a 3- and 6- month period when compared to a usual care control group. Specific Aim 4. Explore and interpret the statistical results obtained in the first quantitative phase to help explain why participants who scored in the lower and upper quartiles on caregiver burden/stress were impacted or not by the usefulness of the intervention. Successful design, delivery, and evaluation of the SCP Plus will lead to a cost effective, sustainable, and accessible intervention that can be implemented in other Senior Companion programs across the country, thus maximizing its impact as a community-based program to address the needs of ADRD caregivers.

PUBLIC HEALTH RELEVANCE: The Senior Companion Program Plus (SCP Plus): A Psychoeducational Intervention for African American Dementia Caregivers Project Narrative Utilizing the Senior Companion Program as a platform for a dementia caregiving intervention offers an innovative approach for providing affordable services and supports in a community setting and is potentially sustainable as it expands the services of an existing program. The Senior Companion Program (SCP Plus) will advance the scientific knowledge about how dementia caregivers from minority populations can build coping skills and protective factors through engagement with lay health care providers such as Senior Companions. Implementation of the SCP Plus will increase insight into the sociocultural experiences of dementia caregivers and will enhance the ability of community-based providers to meet the unique needs of minority caregiver populations.

CRITIQUE 1

Significance: 4
Investigator(s): 1
Innovation: 4
Approach: 4
Environment: 1

Overall Impact: The investigative team for this resubmitted R-15 application proposes to utilize a randomized sequential 2-group mixed methods design for this 3-year study to examine the effects of an in-home intervention utilizing Senior Companions (SCP) to deliver a psychoeducational intervention (SCP Plus) to 57 treatment group community-residing caregivers of African American (AA) older adults diagnosed with Alzheimer's Disease and Related Disorders (ADRD). Caregiver outcomes include reduced stress and burden, improved coping skills, and satisfaction with social support. The SCP program has been adapted from a previously tested intervention using trained lay companions to deliver education in the home, and will be delivered across 6 sites in 3 states. The potential impact of the proposed study is supported by the potential for the SCP Plus treatment to improve outcomes for family caregivers, notably at-risk AA caregivers. However, this potential impact is weakened by lack of clearly defined additive aspects of the proposed study beyond the current well-tested and disseminated caregiver interventions, some of which utilize racially matched lay interventionists. The study has potential to increase the research environment at the sponsoring university although involvement of the 3 undergraduate students as site managers for the remote sites is not clearly defined. Culturally sensitive aspects of the treatment are also not clearly integrated. The PI has a record of involvement in research with caregiving populations and with graduate students. A strong research team has been assembled, including expert consultation from Dr. Gaugler. Designing treatments specifically for racial/ethnic minorities is not essentially innovative, given the proliferation of culturally specific treatments over the past decade, with cited references to support this innovation being >10 years. Racially matched senior companions have also been previously utilized to deliver targeted treatments. Scientific rigor is evident throughout the research methods including the sampling plan, attention to treatment fidelity, detailed training procedures, and well-supported outcome measures. The 6 clinical sites will yield close to 500 Senior Companions across the sites, increasing the likelihood of recruiting an adequate interventionist sample. Some moderate and minor weaknesses in the research methods were identified, including lack of a true time-controlled comparison group and some human subjects concerns. The environment at the University of Texas appears adequate to support the proposed study. The investigators have adequately addressed the majority of weaknesses identified in the previous review. Overall, some evidence for potential impact, a strong research team, and scientific rigor in some of the proposed research methods provides some support for this study, with support being lessened by weaknesses in the potential impact and innovations, and minor to moderate methodological weaknesses.

1. Significance:

Strengths

- The potential impact of the proposed study primarily rests in the intent to improve health-related and quality of life outcomes for AA family caregivers of persons with ADRD.
- Utilizing existing Senior Companion programs increases the likelihood of successfully carrying out the proposed study.
- The study has potential to enrich the research environment at the sponsoring university.

Weaknesses

- The scientific rationale supporting the need for further research testing caregiver support and education interventions lacks detail and does not represent the breadth of well-tested treatments.
- Some in-home caregiver treatments utilize racially matched lay interventionists, with no clearly defined additive aspects of the proposed study.
- Potential impact is further weakened by lack of a plan to assure the treatment is culturally sensitive.

2. Investigator(s):

Strengths

- The PI has a record of involvement in research with caregiving populations, including persons with ADRD and with graduate students.
- A strong research team has been assembled, including expert consultation from Dr. Gaugler.
- A biostatistician has been added to the study team, providing needed expertise in data analytic approaches.

Weaknesses

- None noted.

3. Innovation:

Strengths

- Not essentially innovative.

Weaknesses

- Designing and testing treatments specifically for racial/ethnic minorities is not essentially innovative, given the proliferation of culturally specific treatments over the past decade, with cited references to support this innovation being >10 years.
- Senior companions have also been previously utilized to deliver targeted treatments within the home setting, with racial matching often being inherent in these studies.

4. Approach:

Strengths

- Scientific rigor is evident throughout the research methods including the sampling plan, attention to treatment fidelity, detailed SC training procedures, and well-supported outcome measures.
- Sex as a biological variable was indirectly addressed as a covariate in the proposed data analyses.

- Blinding of assessors to treatment condition lessens the chance of bias.
- The 6 clinical sites will yield close to 500 Senior Companions across the sites, increasing the likelihood of recruiting an adequate interventionist sample.

Weaknesses

- No attention-control component is included for the comparison group.
- Methods for controlling for possible contamination of groups through data analytic approaches are not specified and are not inherently clear (p.53) as written.
- Training for data collectors (interviewers?) and student research assistants is not specified, raising concerns regarding consistent data collection across study sites and over time.
- The power analysis is somewhat weak and it appears to be based on 114 subjects/group rather than the proposed 57 subjects/group.
- While this study is proposed as a mixed methods study, specific approaches to utilize the qualitative findings to inform the quantitative findings are not included, decreasing the likelihood that a true mixed methods approach will be realized.

5. Environment:

Strengths

- The environment at the University of Texas appears adequate to support the proposed study.

Weaknesses

- None noted.

Protections for Human Subjects:

Unacceptable Risks and/or Inadequate Protections

- Some human subjects' concerns are evident, as data will be collected from the Senior Companions, although the consent process is unclear.

Data and Safety Monitoring Plan (Applicable for Clinical Trials Only):

Acceptable

- A D&SM plan is included.

Inclusion of Women, Minorities and Children:

- Sex/Gender: Distribution justified scientifically
- Race/Ethnicity: Distribution justified scientifically
- For NIH-Defined Phase III trials, Plans for valid design and analysis: Not applicable
- Inclusion/Exclusion of Children under 18: Excluding ages <18; justified scientifically
- Descriptions of gender in the target population are provided.

Vertebrate Animals:

Not Applicable (No Vertebrate Animals)

Biohazards:

Not Applicable (No Biohazards)

Resubmission:

- The investigators have addressed the weaknesses identified in the preliminary review.

Applications from Foreign Organizations:

Not Applicable (No Foreign Organizations)

Select Agents:

Not Applicable (No Select Agents)

Resource Sharing Plans:

Not Applicable (No Relevant Resources)

Authentication of Key Biological and/or Chemical Resources:

Not Applicable (No Relevant Resources)

Budget and Period of Support:

Recommend as Requested

CRITIQUE 2

Significance: 1
Investigator(s): 1
Innovation: 3
Approach: 2
Environment: 1

Overall Impact: The proposed project has high public health relevance and will provide a focused context for education and training of students. There are scientific gaps in knowledge regarding how best to address the growing problem of Alzheimer's disease and related dementia (ADRD) that impacts African American patients and their caregivers. The study is responsive to national recommendations. There is a strong scientific premise for the study and it builds upon an existing program and implementation infrastructure. The proposed RCT design is well-described and the implementation plan builds upon a national model and a pilot study led by the investigators. The project will be co-led by new investigators with complementary areas of expertise. The PIs are joined by experienced investigators. The commitment and experience with training students appears reasonable and there is a track record of integrating students within research projects. Overall this is a strong project with identified collaborators that has multiple opportunities to engage students.

1. Significance:

Strengths

- Alzheimer's Disease has a major economic and social impact in the US. AD is debilitating for the person diagnosed and caregivers often face a host of challenges including depression, anxiety, and health impairments in comparison to non-caregivers.
- Patients who reside in the community often receive care from caregivers who provide uncompensated care and who suffer from poor psychosocial and psychological health

challenges. A strong rationale is provided for peer-led psychoeducational models to support AD patients and their caregivers. Additionally, information is provided that supports the need to assess cultural factors and/or contextual factors that may be relevant for African Americans. There is a strong scientific premise that African Americans have differential patterns of care-seeking.

- African American older adults are at higher risk for developing late-onset AD compared to whites and their caregivers generally have poorer health and health behaviors than their white caregiver counterparts suggesting a need for interventions to target this subgroup.
- The intervention leverages an existing national caregiving program in three states to integrate a psycho-educational intervention, which should enhance dissemination and/or sustainability.
- Provision of the intervention in the home may have potential for greater impact than an out of home intervention.

Weaknesses

- No score-driving weaknesses

2. Investigator(s):

Strengths

- Two investigators will serve as MPIs on the project. Dr. Fields' work is focused on cultural issues in caregiving for individuals with dementia. She led a pilot study that informed the proposed study. While at UT, Dr. Fields has had 8 students work with her on research projects over the past 4 years. Dr. Fields is a new investigator focused on cultural issues in caregiving for individuals with dementia. In addition to clinical experience serving persons with dementia she led a pilot study of the SCP Plus. Dr. Xu has expertise in family gerontology and collaborated with Dr. Fields on the SCP pilot project.
- Co-investigators are independent and experienced investigators (e.g., Drs. Cipher, Gaugler).

Weaknesses

- No score-driving weaknesses.

3. Innovation:

Strengths

- Innovative components of solely focusing on African American caregivers, inclusion of culturally relevant factors (e.g., religious coping), delivery of the intervention in the home setting and integration of a qualitative component for persons in specified quartiles may inform intervention adaptation.

Weaknesses

- The SCP program appears well-developed and wide-spread which limits innovation.

4. Approach:

Strengths

- A rigorous research design is proposed (RCT). The inclusion of multiple research sites across three states may add to generalizability and also allow for variation in sample characteristics.
- The SCP plus is based on a dementia care giving program and the modules of the Senior Companion Program are well detailed.

- The sociocultural stress and coping model will be employed as the conceptual model to guide the intervention and the study. Key variables relevant to model and measures are well-integrated (coping style, social support, well-being)
- The setting and process for engaging peer companions is well described.
- The PIs and intervention staff will monitor the quality of intervention delivery using various methods that includes a checklist.
- The plan for recruitment and hiring of students is described.
- Over the course of the project undergraduate, graduate and doctoral students will be integrated as research assistants. A process is outlined for selecting students based on performance in key courses. Specific roles of the students are outlined.
- The proposed training activities and level of integration with the research projects are likely to be beneficial to students.

Weaknesses

- There was some question about the integration of the two phases of the qualitative data collection although there is detail on the content analysis.

5. Environment:

Strengths

- The interventions will be nested within an existing national SCP that trains and deploys peer companions to families that include a person diagnosed with AD.
- The environment appears well suited for the proposed research.
- Multiple sites and programs are included that have the capacity to implement the project.

Weaknesses

- No score-driving weaknesses.

Protections for Human Subjects:

Acceptable Risks and/or Adequate Protections

- Processes for maintaining confidentiality and implementing participant protections is described in the application.

Data and Safety Monitoring Plan (Applicable for Clinical Trials Only):

Acceptable

- o Plans to monitor and report adverse events are described.

Inclusion of Women, Minorities and Children:

- Sex/Gender: Distribution justified scientifically
- Race/Ethnicity: Distribution justified scientifically
- For NIH-Defined Phase III trials, Plans for valid design and analysis: Not applicable
- Inclusion/Exclusion of Children under 18: Excluding ages <18; justified scientifically
- Children are excluded, males and females are included and the study is focused on African American caregivers.

Vertebrate Animals:

Not Applicable (No Vertebrate Animals)

Biohazards:

Not Applicable (No Biohazards)

Resubmission:

- More detail on the explanatory sequential mixed method design is included. The fidelity plan was revised.

Applications from Foreign Organizations:

Not Applicable (No Foreign Organizations)

Select Agents:

Not Applicable (No Select Agents)

Resource Sharing Plans:

Acceptable

Authentication of Key Biological and/or Chemical Resources:

Not Applicable (No Relevant Resources)

Budget and Period of Support:

Recommend as Requested

CRITIQUE 3

Significance: 5
Investigator(s): 4
Innovation: 3
Approach: 6
Environment: 3

Overall Impact: The purpose of this study is to evaluate "SCP Plus"--a 9 module psychoeducation intervention delivered by lay providers (e.g. peers referred to as Senior Companions)--on outcomes of caregiver knowledge, burden, wellbeing and coping at 3- and 6-months when compared to a usual care control group. The intervention will be tested across several senior service agencies with existing SCP programs in Texas, Louisiana and Arkansas. This R-15 project will also provide mentored research learning experiences to social work students in community-based research with high risk, high need populations. Strengths of the application include the use of RCT design, a conceptually grounded intervention and the use of well-validated measures. Weaknesses are the lack of evidence supporting the scientific premise, the limited generalizability due to restricting sample to African American caregivers who have access to and participate in SCP programs, lack of details on how the intervention is culturally tailored to address the needs of low income African American caregivers, and missing details on data collection procedures. Thus, despite the importance of this topic, the moderate level

weaknesses limit the potential impact of the study on advancing interventions to support dementia caregivers.

1. Significance:

Strengths

- The study addresses an important problem-- the need for culturally sensitive psychoeducational interventions to support family caregivers of persons with dementia, especially those in underserved communities.

Weaknesses

- The scientific premise of the study is that trained peer volunteers without any prior clinical background or expertise can provide a 9-week psychoeducational intervention that improves coping, social support and wellbeing in a high-risk, high need population of low income African American dementia caregivers. The evidence provided to support this premise is weak.
- It remains unclear why the investigators are solely focusing on low income African American caregivers in their study sample. There is little information provided on how SCP Plus is culturally adapted to meet the specific needs of this population of dementia caregivers, and moreover how lay providers are prepared to address these complex needs.

2. Investigator(s):

Strengths

- Drs. Fields and Xu have beginning programs of research in psychoeducational interventions for minority dementia caregivers They bring established record of mentoring undergraduate and graduate students and have successfully collaborated in the past.
- Dr Gaugler is an expert in dementia caregiving interventions. He will provide 27 hours per year of consultation on implementation, data collection, analysis and dissemination.
- Multi PI plan is appropriate

Weaknesses

- Dr. Williams is an Assistant professor at UVA. It is unclear how her expertise will be utilized (1 day per year) to “enhance recruitment, data collection and analysis” specific to the African America participants given that her bio suggests that agencies will need to “reach out for consultative support if their site is having challenges”.
- No mention of other existing resources available to investigators to address recruitment challenges or the other culturally tailoring that will be necessary to adopt this intervention specifically for low income African American dementia caregivers.

3. Innovation:

Strengths

- Builds on existing SCP program by adding a lay provider component to bring social support into the home.

Weaknesses

- No score-driving weaknesses noted.

4. Approach:

Strengths

- Use of RCT design to enhance rigor
- Use of well established, well validated measures of caregiver outcomes, mapped to conceptual framework
- The intervention is conceptually grounded in the theory of stress and coping.
- Up to six social work students will receive research training as part of the proposed research study
- Sex is considered as a variable-- appropriate sub analysis is proposed to study gender-based differences.

Weaknesses

- Generalizability of the findings is limited since sample population is caregivers enrolled in SCP program. The majority of dementia caregivers, and particularly African American caregivers, are not receiving supportive services. The investigators do not address this significant limitation
- The investigators do not outline specific approaches to engage the low-income African-American community (other than letters of support from agencies)
- No procedures detailing data collection such as training and monitoring of data collectors, process for data collection, or blinding of data collectors.
- No attention in analysis to controlling for likely differences across study sites/States.

5. Environment:

Strengths

- Letters of support from sites identifying Senior Companions and eligible caregivers are provided.

Weaknesses

- While rich resources are available at UT-Arlington to enhance the research and mentorship activities planned, there is no discussion of how these resources will be engaged to provide additional research training and mentorship to students.

Protections for Human Subjects:

Acceptable Risks and/or Adequate Protections

- Sufficient attention to protection of human subjects

Data and Safety Monitoring Plan (Applicable for Clinical Trials Only):

No concerns noted

Inclusion of Women, Minorities and Children:

- Sex/Gender: Distribution justified scientifically
- Race/Ethnicity: Distribution justified scientifically
- For NIH-Defined Phase III trials, Plans for valid design and analysis: Not applicable
- Inclusion/Exclusion of Children under 18: Excluding ages <18; justified scientifically

Resubmission:

- Only partially responsive to prior reviews and concerns remain that were raised in the prior review. For example, added biostatistician to study team but does not address the need to capitalize on other rich research resources available at UT, rather relies on 2 external consultants. The external validity/generalizability remains limited given the sample population of persons receiving services.

Budget and Period of Support:

Recommend as Requested

THE FOLLOWING SECTIONS WERE PREPARED BY THE SCIENTIFIC REVIEW OFFICER TO SUMMARIZE THE OUTCOME OF DISCUSSIONS OF THE REVIEW COMMITTEE, OR REVIEWERS' WRITTEN CRITIQUES, ON THE FOLLOWING ISSUES:

PROTECTION OF HUMAN SUBJECTS: UNACCEPTABLE

- A reviewer noted that consenting of Senior Companions requires clarification.

INCLUSION OF WOMEN PLAN: ACCEPTABLE

INCLUSION OF MINORITIES PLAN: ACCEPTABLE

INCLUSION OF CHILDREN PLAN: ACCEPTABLE

COMMITTEE BUDGET RECOMMENDATIONS: The budget was recommended as requested.

Revision: It was noted that a draft version was uploaded on March 1st. Final version uploaded on March 5th.

Footnotes for 1 R15 AG058182-01A1; PI Name: Fields, Noelle

+ Derived from the range of percentile values calculated for the study section that reviewed this application.

NIH has modified its policy regarding the receipt of resubmissions (amended applications). See Guide Notice NOT-OD-14-074 at http://grants.nih.gov/grants/guide/notice-files/NOT-OD-14-074.html. The impact/priority score is calculated after discussion of an application by averaging the overall scores (1-9) given by all voting reviewers on the committee and multiplying by 10. The criterion scores are submitted prior to the meeting by the individual reviewers assigned to an application, and are not discussed specifically at the review meeting or calculated into the overall impact score. Some applications also receive a percentile ranking. For details on the review process, see http://grants.nih.gov/grants/peer_review_process.htm#scoring.

MEETING ROSTER

Center for Scientific Review Special Emphasis Panel
CENTER FOR SCIENTIFIC REVIEW
Nursing and Related Clinical Sciences
ZRG1 NRCS-V (08)
02/08/2018 - 02/09/2018

Notice of NIH Policy to All Applicants: Meeting rosters are provided for information purposes only. Applicant investigators and institutional officials must not communicate directly with study section members about an application before or after the review. Failure to observe this policy will create a serious breach of integrity in the peer review process, and may lead to actions outlined in NOT-OD-14-073 at https://grants.nih.gov/grants/guide/notice-files/NOT-OD-14-073.html and NOT-OD-15-106 at https://grants.nih.gov/grants/guide/notice-files/NOT-OD-15-106.html, including removal of the application from immediate review.

CHAIRPERSON(S)

DOZIER, ANN MARIE, PHD, RN
PROFESSOR AND CHAIR
DEPARTMENT OF PUBLIC HEALTH SCIENCES
UNIVERSITY OF ROCHESTER
ROCHESTER, NY 14642

MEMBERS

ARCOLEO, KIMBERLY JOAN, PHD
ASSOCIATE PROFESSOR
ASSOCIATE DEAN FOR RESEARCH
SCHOOL OF NURSING
UNIVERSITY OF ROCHESTER
ROCHESTER, NY 14642

BERGER, ANN M, PHD, BSN
PROFESSOR
DOROTHY HODGES OLSON ENDOWED CHAIR IN NURSING
ADVANCE PRACTICE NURSE, ONCOLOGY
COLLEGE OF NURSING
UNIVERSITY OF NEBRASKA MEDICAL CENTER
OMAHA, NE 68198-5330

BONATO, PAOLO, PHD
ASSOCIATE PROFESSOR
SPAUDLING REHABILITATION HOSPITAL
MOTION ANALYSIS LABORATORY
HARVARD MEDICAL SCHOOL
BOSTON, MA 02114

BRITTAIN, KELLY, RN, PHD
ASSOCIATE PROFESSOR
NURSING EDUCATION AND RESEARCH
COLLEGE OF NURSING
MICHIGAN STATE UNIVERSITY
EAST LANSING, MI 48824

BURGENER, SANDRA C, PHD, RN, FAAN
ASSOCIATE PROFESSOR EMERITA
DEPARTMENT OF BIOBEHAVIORAL HEALTH NURSING
UNIVERSITY OF ILLINOIS AT URBANA-CHAMPAIGN
URBANA, IL 46202

DAVIS, CLAUDIA M, PHD
ASSOCIATE PROFESSOR
CENTER FOR HEALTH DISPARITIES RESEARCH & TRAINING
DEPARTMENT OF NURSING
COLLEGE OF NATURAL SCIENCES
CALIFORNIA STATE UNIVERSITY SAN BERNADINO
MORENO VALLEY, CA 92557

DOUGHERTY, CYNTHIA M, BSN, PHD
PROFESSOR
DEPARTMENT OF MEDICINE
UNIVERSITY OF WASHINGTON
SEATTLE, WA 98108-1595

EDLUND, MARK J, PHD, MD
SENIOR RESEARCH SCIENTIST
BEHAVIORAL HEALTH EPIDEMIOLOGY PROGRAM
RTI INTERNATIONAL
RESEARCH TRIANGLE PARK, NC 27709

FENTON, SUSAN HRACHOVY, PHD
ASSOCIATE DEAN FOR ACADEMIC AFFAIRS
SCHOOL OF BIOMEDICAL INFORMATICS
UNIVERSITY OF TEXAS
HOUSTON, TX 77030

FINLEY, MARGARET ANNE, PHD
ASSOCIATE PROFESSOR
DEPT OF PHYSICAL THERAPY AND REHAB SCIENCE
DREXEL UNIVERSITY
PHILADELPHIA, PA 19102

GEORGE, MAUREEN, PHD, RN
ASSOCIATE PROFESSOR
SCHOOL OF NURSING
COLUMBIA UNIVERSITY
NEW YORK CITY, NY 10032

GIBSON, ROBERT WILLIAM, PHD
PROFESSOR AND DIRECTOR OF RESEARCH
DEPARTMENT OF EMERGENCY MEDICINE
MEDICAL COLLEGE OF GEORGIA
AUGUSTA UNIVERSITY
AUGUSTA, GA 30912

GLICK, SUSAN B, MD
ASSOCIATE PROFESSOR
INTERNAL MEDICINE
RUSH MEDICAL COLLEGE
CHICAGO, IL 60612

HAQUE, REINA, PHD
RESEARCH SCIENTIST III & SCIENTIFIC ADVISOR, CANCER REGISTRY
DEPARTMENT OF RESEARCH & EVALUATION
KAISER PERMANENTE SOUTHERN CALIFORNIA
PASADENA, CA 91101

HEPBURN, KEN W, PHD
PROFESSOR
DIRECTOR OF GRADUATE STUDIES
CO-CHAIR, DEPARTMENT OF ADULT AND ELDER HEALTH
NELL HODGSON WOODRUFF SCHOOL OF NURSING
EMORY UNIVERSITY
ATLANTA, GA 30322

HODGSON, NANCY A, BSN, PHD, RN
ASSOCIATE PROFESSOR
DEPARTMENT OF BIOBEHAVIORAL HEALTH SCIENCES
UNIVERSITY OF PENNSYLVANIA SCHOOL OF NURSING
PHILADELPHIA, PA 19104

HUDSON, TERESA JO, PHD
DIRECTOR
DIVISION OF HEALTH SERVICES RESEARCH
PSYCHIATRIC RESEARCH INSTITUTE
UNIVERSITY OF ARKANSAS FOR MEDICAL SCIENCES
LITTLE ROCK, AK 72205

JACOB, EUFEMIA, BSN, PHD, RN
ASSOCIATE PROFESSOR
UCLA SCHOOL OF NURSING
DEPARTMENT OF PEDIATRICS
700 TIVERTON AVENUE
LOS ANGELES, CA 90095

JONES, RANDY ALLEN, BSN, PHD, RN
ASSOCIATE PROFESSOR
SCHOOL OF NURSING
UNIVERSITY OF VIRGINIA
CHARLOTTESVILLE, VA 22904-4195

LAI, JIN-SHEI, PHD
PROFESSSOR
DEPARTMENT OF MEDICAL SOCIAL SCIENCES
FEINBERG SCHOOL OF MEDICINE
NORTHWESTERN UNIVERSITY
CHICAGO, IL 60611

MASKO, MEGANNE KATHLEEN, PHD
ASSISTANT PROFESSOR
MUSIC AND ARTS TECHNOLOGY
INDIANA UNIVERSITY-PURDUE UNIVERSITY
INDIANAPOLIS, IN 46202

NESS, KIRSTEN KIMBERLIE, PHD
FULL MEMBER
DEPARTMENT OF EPIDEMIOLOGY AND CANCER CONTROL
SAINT JUDE CHILDREN'S RESEARCH HOSPITAL
MEMPHIS, TN 38105

PIATT, GRETCHEN A, PHD
ASSOCIATE PROFESSOR
DEPARTMENT OF LEARNING HEALTH SCIENCES
UNIVERSITY OF MICHIGAN MEDICAL SCHOOL
ANN ARBOR, MI 48109

RIDNER, SHEILA H, PHD, BSN
MARTHA RIVERS INGRAM PROFESSOR
AND DIRECTOR OF GRADUATE
STUDIES PHD NURSING SCIENCE
SCHOOL OF NURSING
VANDERBILT UNIVERSITY
NASHVILLE, TN 37240

RUNNING, ALICE FAY, PHD
PROFESSOR
COLLEGE OF NURSING
MONTANA STATE UNIVERSITY
BOZEMAN, MT 59717

SCHROECK, FLORIAN R, MD
SECTION CHIEF OF UROLOGY
ASSISTANT PROFESSOR OF SURGERY
WHITE RIVER JUNCTION VETERANS AFFAIRS MEDICAL CTR
DARTMOUTH HITCHCOCK MEDICAL CENTER
LEBANON, NH 03756

SECOR, ERIC RICHARD JR, ND, PHD
ASSOCIATE DIRECTOR
DIVISION OF INTEGRATIVE MEDICINE
DEPARTMENT OF MEDICINE
HARTFORD HOSPITAL
UNIVERSITY OF CONNECTICUT SCHOOL OF MEDICINE
HARTFORD, CT 06102

SHEPPARD, VANESSA B, PHD
ASSOCIATE PROFESSOR
ASSOCIATE DIRECTOR
HEALTH DISPARITIES RESEARCH
MASSEY CANCER CENTER
VIRGINIA COMMONWEALTH UNIVERSITY
RICHMOND, VA 23298

SHERWOOD, NANCY E, PHD
ASSOCIATE PROFESSOR
DIVISION OF EPIDEMIOLOGY AND COMMUNITY HEALTH
UNIVERSITY OF MINNESOTA
MINNEAPOLIS, MN 55455

SILLS, MARION RUTH, MD
ASSOCIATE PROFESSOR
CO-DIRECTOR, TL1 PROGRAM, CCTSI
PEDIATRICS-EMERGENCY MEDICINE
SCHOOL OF MEDECINE
UNIVERSITY OF COLORADO
AURORA, CO 80045

SZALACHA, LAURA A, EDD
PROFESSOR
COLLEGE OF NURSING
UNIVERSITY OF ARIZONA
TUCSON, AZ 85721

TUROK, DAVID, MD
ASSOCIATE PROFESSOR
DEPARTMENT OF OBSTETRICS AND GYNECOLOGY
UNIVERSITY OF UTAH
SALT LAKE CITY, UT 84132

UPHOLD, CONSTANCE R, PHD
ASSOCIATE DIRECTOR
IMPLEMENTATION AND OUTCOMES RES
GERIATRIC RESEARCH EDUCATION CLINICAL CENTER
GAINESVILLE VA MEDICAL CENTER
UNIVERSITY OF FLORIDA COLLEGE OF MEDICINE
GAINESVILLE, FL 32608

VOSS, JOACHIM G, PHD, RN
PROFESSOR AND ENDOWED DIRECTOR
SARA COLE HIRSH CENTER FOR EVIDENCE BASED PRACTICE
FRANCES PAYNE BOLTON
SCHOOL OF NURSING
CASE WESTERN RESERVE UNIVERSITY
CLEVELAND, OH 44106

WANG, DONGWEN, PHD
PROFESSOR
DEPARTMENT OF BIOMEDICAL INFORMATICS
ARIZONA STATE UNIVERSITY
SCOTTSDALE, AZ 85259

WEISS, SANDRA JEAN, PHD, DNSC, FAAN, RN
PROFESSOR AND ESCHBACH ENDOWED CHAIR
SCHOOL OF NURSING COMMUNITY HEALTH SYSTEMS
SCHOOL OF NURSING
UNIVERSITY OF CALIFORNIA, SAN FRANCISCO
SAN FRANCISCO, CA 94143

WELLS, KRISTEN JENNIFER, PHD
ASSOCIATE PROFESSOR
DEPARTMENT OF PSYCHOLOGY
SAN DIEGO STATE UNIVERSITY
SAN DIEGO, CA 92120

WILLIAMS, KAREN PATRICIA, PHD
DISTINGUISHED PROFESSOR OF WOMEN'S HEALTH, DIRECTOR
CENTER FOR WOMEN, CHILDREN AND YOUTH
COLLEGE OF NURSING
OHIO STATE UNIVERSITY
COLUMBUS, OH 43210

WITTENBERG, ELAINE M, PHD
ASSOCIATE PROFESSOR
DEPARTMENT OF COMMUNICATION STUDIES
CALIFORNIA STATE UNIVERSITY, LOS ANGELES
LOS ANGELES, CA 90032

MAIL REVIEWER(S)

ALVAREZ, OFELIA AMPARO, MD
PROFESSOR OF CLINICAL PEDIATRICS
DEPARTMENT OF PEDIATRICS
UNIVERSITY OF MIAMI HEALTH SYSTEM
MIAMI, FL 33136

GALARRAGA, OMAR, PHD
ASSISTANT PROFESSOR
DEPARTMENT OF HEALTH SERVICES, POLICY & PRACTICE
BROWN UNIVERSITY
PROVIDENCE, RI 02912

KEMPER, ALEX R, MD
PROFESSOR
NATIONWIDE CHILDREN'S HOSPITAL
THE OHIO STATE UNIVERSITY
COLUMBUS, OH 43205

WILLIAMS, EDITH MARIE, PHD
RESEARCH ASSISTANT PROFESSOR
DEPARTMENT OF EPIDEMIOLOGY AND BIOSTATISTICS
UNIVERSITY OF SOUTH CAROLINA AT COLUMBIA
COLUMBIA, SC 29210

SCIENTIFIC REVIEW OFFICER

HARE, MARTHA L, RN, PHD
SCIENTIFIC REVIEW OFFICER
CENTER FOR SCIENTIFIC REVIEW
NATIONAL INSTITUTES OF HEALTH
BETHESDA, MD 20892

EXTRAMURAL SUPPORT ASSISTANT

JONES, BELINDA
EXTRAMURAL SUPPORT ASSISTANT
CENTER FOR SCIENTIFIC REVIEW
NATIONAL INSTITUTES OF HEALTH
BETHESDA, MD 20892

Consultants are required to absent themselves from the room during the review of any application if their presence would constitute or appear to constitute a conflict of interest.
